# Bone Marrow Niches and Tumour Cells: Lights and Shadows of a Mutual Relationship

**DOI:** 10.3389/fimmu.2022.884024

**Published:** 2022-05-06

**Authors:** Valentina Granata, Laura Crisafulli, Claudia Nastasi, Francesca Ficara, Cristina Sobacchi

**Affiliations:** ^1^ IRCCS Humanitas Research Hospital, Milan, Italy; ^2^ Milan Unit, CNR-IRGB, Milan, Italy; ^3^ Laboratory of Cancer Pharmacology, Department of Oncology, IRCCS Mario Negri Pharmacological Research Institute, Milan, Italy

**Keywords:** bone marrow niches, hematopoietic stem cells (HSCs), MSCs, myeloid neoplasms, metastasis, targeted therapy, RANKL, JAK2

## Abstract

The bone marrow (BM) niche is the spatial structure within the intra-trabecular spaces of spongious bones and of the cavity of long bones where adult haematopoietic stem cells (HSCs) maintain their undifferentiated and cellular self-renewal state through the intervention of vascular and nervous networks, metabolic pathways, transcriptional and epigenetic regulators, and humoral signals. Within the niche, HSCs interact with various cell types such as osteoblasts, endothelial cells, macrophages, and mesenchymal stromal cells (MSCs), which maintain HSCs in a quiescent state or sustain their proliferation, differentiation, and trafficking, depending on body needs. In physiological conditions, the BM niche permits the daily production of all the blood and immune cells and their admittance/ingress/progression into the bloodstream. However, disruption of this delicate microenvironment promotes the initiation and progression of malignancies such as those included in the spectrum of myeloid neoplasms, also favouring resistance to pharmacological therapies. Alterations in the MSC population and in the crosstalk with HSCs owing to tumour-derived factors contribute to the formation of a malignant niche. On the other hand, cells of the BM microenvironment cooperate in creating a unique milieu favouring metastasization of distant tumours into the bone. In this framework, the pro-tumorigenic role of MSCs is well-documented, and few evidence suggest also an anti-tumorigenic effect. Here we will review recent advances regarding the BM niche composition and functionality in normal and in malignant conditions, as well as the therapeutic implications of the interplay between its diverse cellular components and malignant cells.

## Introduction

Bone marrow (BM) niches are specialized microenvironments within bones where supportive cells forming the cellular niche allow the maintenance and differentiation of haematopoietic and mesenchymal stem cells (HSCs and MSCs, respectively) (1, 2). The cellular characterization of these complex microenvironments has been achieved mainly by means of elaborated genetic approaches targeting selected candidate factors, despite limitations of specificity inherent to this strategy, resulting in controversial conclusions. More recently, the combined application of state-of-the-art technologies including high resolution imaging, single-cell RNA sequencing (scRNAseq) and spatially resolved transcriptomics has led to an unprecedented insight into the molecular, cellular, and spatial organization of BM niches, and hierarchical structures and differentiation trajectories therein (3–6). On this basis, sinusoidal, arteriolar and endosteal niches appear to be distinguished not only by their specific location, but also by their unique cellular composition and molecular requirements. Moreover, increasing evidence demonstrate that alterations at different levels in the niche composition are associated with malignancies including myelodysplastic syndromes (MDS) (7) and myeloproliferative neoplasms (MPN) (8), with an additional effect on the osteo-haematopoietic niche deriving from medical treatments (9, 10). Remarkable efforts have been devoted also to translating this basic knowledge, mainly derived from murine models, into the development of relevant *in vitro* platforms to study the human counterpart and to test drugs in a patient-specific setting (11, 12). As a perspective, these tools may also serve to test the inherent therapeutic potential of the various niche components.

This expanding field has been covered by several comprehensive reviews. Here we will give an overview of the interplay between BM niches and tumour cells (TCs), focus preferentially on very last papers, and highlight therapeutic implications and perspectives of this mutual relationship, for the benefit of a wide audience ranging from neophytes to experts.

## Composition of BM Niches

MSC and HSC lineage cells are responsible for the high dynamism of the bone tissue, and shape the BM niches, spatially defined microenvironments classified as endosteal and subendosteal (based on the distance from the inner bone surface, particularly at the metaphyseal spongiosa), arteriolar (close to the main vessels carrying blood into the BM), sinusoidal (next to vessels carrying blood out of the BM and forming a wide network within the BMME) and non-vascular (6), based on their location. Overall, niche cell composition is varied, with a distinct array of components in a specific spatial location. Osteoblasts (OBs), chondrocytes and endosteal fibroblasts are present only in the endosteal niche; arteriolar endothelial cells (ECs), smooth muscle cells, arteriolar fibroblasts localize to the arteriolar niche; a newly identified subset of CXCL12-abundant reticular (CAR) cells called Osteo-CARs, displaying a high expression of CXCL12 and osteolineage genes such as osterix (SP7) and lower leptin receptor (LEPR), localize to arteriolar and non-vascular niches; sinusoidal ECs are found in the sinusoidal and in the (sub-)endosteal niches, in line with sinusoids’ elongation through the entire BM cavity. In the proximity of sinusoids, another CAR subpopulation called Adipo-CAR, characterized by high expression of adipogenic lineage genes, resides, too. Interestingly, this subtle dissection of the BM cellular niche has highlighted the heterogeneity of the CAR cell population, specialized MSCs essential for the HSC maintenance and control at many developmental stages, recently isolated also from human adult BM (13). On the other hand, consensus on the panel of protein markers unique for murine and human MSCs is still lacking (see section *A Role for MSCs in MPN*) and establishing the relationship between MSC subsets described by various recent works is not straightforward (14).

At steady state, HSCs are mostly quiescent and located in perisinusoidal niches, while periarteriolar niches are important mainly for lymphopoiesis (15–17). Committed progenitors likely also exploit dedicated sinusoidal niches, as revealed by elegant approaches of *in situ* mapping, delineating an atlas of spatially and functionally distinct niches (6). For what pertains to endosteal niches, and the contribution of fully differentiated skeletal cells to HSC maintenance, opposed pieces of evidence are present in the literature with respect to osteoclasts (OCs) (18, 19); OBs have been the first cell population reported to support HSCs (20, 21), but later studies have clearly demonstrated that the major role is played by CAR cells, not by OBs (22–24), so OB contribution has yet to be clarified. Finally, osteocytes might influence specifically myelopoiesis by means of secreted molecules (25).

A plethora of supportive factors are provided in the niches (**Table 1**). Recent evidence showed that the key niche factors SCF and CXCL12 can be modulated by the Caspase3/NLRP3 signaling, which extends understanding of regulatory mechanisms influencing haematopoiesis (40). Of note, the same molecular cue presented by diverse cells in BM niches may serve different functions in each compartment. For example, Himburg and colleagues recently demonstrated that the heparin-binding growth factor pleiotrophin, already known to promote HSC expansion *in vitro* and HSC regeneration *in vivo*, must be provided by LepR^+^ BM stromal cells for HSC maintenance during steady-state haematopoiesis, and by ECs for HSC regeneration after injury (irradiation) (32). Similar restrictions in growth factor provision apply to committed haematopoietic progenitors: for example, the maintenance of the pool of c-Kit^+^ haematopoietic progenitors requires (among other factors) SCF supply from LepR^+^ BM stromal cells and not from ECs (41). On the other hand, the same factor may elicit different effects on haematopoietic stem and progenitor cells (HSPCs) versus more downstream committed progenitors. For example, the secreted RNase angiogenin, expressed by BM osteolineage cells, has been recently demonstrated to restrict proliferation of primitive HSPCs, on one hand, and stimulate proliferation of myeloid-restricted progenitors, on the other, owing to a differential effect on RNA processing in the two subsets (42). Overall, this further demonstrates the cellular specialization within BM niches.

**Table 1:** Soluble factors in BM niches.

FACTOR	TYPE OF MOLECULE	MAIN ROLE IN THE BM NICHE	REF
**CXCL12**	Chemokine secreted by OBs, ECs, CAR cells and MSCs	HSC self-renewal and BM retention	(26)
**SCF**	Cytokine secreted by OBs, ECs, and MSCs	Stimulation and self-renewal of HSC	(27)
**IL-6**	Cytokine secreted by OBs	HSC proliferation *in vitro*, regulation of inflammation	(28)
**OSM**	Cytokine secreted by OBs and macrophages	Regulation and mobilization of HPSCs	(29)
**TPO**	Cytokine secreted mostly by megakaryocytes, OBs	Maintenance and self-renewal of HSCs, HSC homing	(30)
**G-CSF**	Growth factor secreted by OBs	Proliferation and differentiation of HSCs, HSC mobilization	(31)
**PTN**	Growth factor secreted by sinusoidal ECs, LepR+ perivascular cells	HSC self-renewal and HSC proliferation	(32)
**FGF2**	Growth factor secreted by OBs	Support HSC homeostasis	(33)
**Angpt**	Growth factor secreted by HSCs, HPSCs, megakaryocytes, and LepR^+^ stromal cells	Regulation of niche regeneration	(34)
**TGF-β**	Growth factor secreted by Schwann cells, megakaryocytes	Maintenance of HSC quiescence	(35)
**JAG1**	Notch-ligand secreted by endothelial cells and osteoblast	Regeneration of BM niche during injury, HSC regulation	(36)
**PGI_2_**	Hormone released by endosteal cells	HSC reconstitution and limits HSC exhaustion	(37)
**OPN**	Glycoprotein produced by MSCs, OBs, ECs	HSC migration and self-renewal	(38)
**VCAM1**	Cell adhesion molecule expressed by ECs, MSCs	HSC maintenance	(39)

## BM Niches as Tumour Cell Factory In Acquired Blood Disorders: A Focus on MPN and MDS

At the end of the 19th century, Stephen Paget postulated the “seed and soil” hypothesis that stated that TC (seeds) need a propitious medium (soil) to establish metastases. This concept can be applied also to malignant cells giving rise to haematological diseases. Most types of blood cancer, including acute and chronic leukaemia, myeloproliferative disorders and MDS, are primarily driven by accumulation of mutations in HSCs or in their progenitors. Growth and survival of the malignant clone is favoured by age-related or inflammation-driven changes in the BM microenvironment (BMME) (43, 44). On the other hand, BMME is affected by signals coming from mutated HSCs, in a bidirectional crosstalk (45).

Myeloproliferative neoplasms (MPN) and MDS are two paradigmatic and opposite examples of diseases caused by mutated HSCs that in turn can alter the surrounding microenvironment, although detailed mechanisms have not been completely defined. MPN include essential thrombocythemia, polycythaemia vera, and primary myelofibrosis, characterized by excess of platelets, erythrocytosis or myelofibrosis, respectively, with increased risk of thrombotic events and of leukemic transformation (46). They are due to mutations occurring at the HSC level in JAK2, MPL or CALR genes, all resulting in unregulated activation of the JAK/STAT pathway, although involvement of other genes (47) or microenvironmental factors contribute to disease initiation or progression.

MDS are a heterogeneous group of acquired clonal disorders of HSCs, characterized by ineffective haematopoiesis, peripheral cytopenia, genetic instability, and high risk of progression to acute myeloid leukaemia (AML) (48). MDS- and MPN-HSPCs display cell-intrinsic dysregulation of innate immune and inflammatory pathways, which in turn have an impact on the surrounding BMME. For example, MDS-HSPCs have aberrantly high expression of TLRs, which activate the adaptive immune system contributing to maintain an inflammatory environment that is detrimental for HSC function, characterized by increased local and systemic levels of IL-6, IL-1β, or type 1 IFN (49). The concomitant presence of chronic inflammation and of increased levels of anti-inflammatory proteins like TGF-β and TNF-α, contribute also to expand BM myeloid-derived suppressor cells (MDSCs), known to dampen T and natural killer (NK) cell anti-tumour activity (50). Other constituents of the BMME that are dysfunctional in MDS or MPN include ECs, within the vascular niche, and Schwann cells. MDS patients display increased BM microvascular density, likely due to the reported secretion of angiogenic growth factors from MDS cells. ECs from MDS patient manifest genetic, transcriptional and epigenetic modifications, along with secretion of supportive myeloid growth factors, further favouring the growth of the malignant clone (51). Regarding Schwann cells, their number is decreased in MPN compared to healthy donors (HDs), while it is markedly higher in MDS patients with severe fibrosis. Despite the relevance for the pathophysiology has not been demonstrated, one hypothesis is that Schwann cells enhance TGF-β activation, which contributes to the suppression of normal haematopoiesis as well as the promotion of BM fibrosis (52).

All the cell populations mentioned above cooperate in creating a unique milieu that favours TC immune evasion and promotes disease progression. Among all the different BMME components, here we will focus on MSCs, which can influence the malignant clone directly, through an altered HSC-supportive capacity, or by exerting immunosuppressive functions on innate and adaptive immunity cells, thus indirectly affecting malignant HSCs by favouring evasion from immunosurveillance.

### A Role for MSCs in MPN

MSCs are multipotent cells able to differentiate into OBs, adipocytes, and chondrocytes. They are commonly characterized by spindle-shape morphology, plastic adherence, *in vitro* trilineage differentiation and expression of surface markers (comprising CD73, CD90, CD105, CD146, CD106, STRO‐1, SSEA‐4, CD49a, CD27, CD146, and LepR as positive markers, and CD45, CD34, CD19, CD14, CD11b, HLA II as negative ones) (53, 54), while tests for single-cell renewal and multipotency are usually omitted, which may raise some concern regarding the actual stemness of these cells. Even recent markers (e.g., LepR) are not specific and label also mature cell types (4). Lastly, Skeletal Stem Cells (SSCs), defined as bone-resident stem cells committed to skeletogenesis and able to recapitulate bone organogenesis *in vivo*, have been more reliably isolated from single-cell suspensions after bone enzymatic digestion (14). With this strategy, two spatially distinct SSC populations have been defined: osteochondral SSCs, giving rise to bone, cartilage and stromal lineage; and perivascular SSCs, displaying also adipogenic potential and HSC supportive capacity (55). This protocol is demanding and not yet routinely adopted. Human MSC characterization according to the criteria described above has highlighted some differences between HD- and MPN-derived MSCs. For example, increased expression of CD90, CD73, and CD44, and lower expression of CD105 has been reported in MPN-MSCs as compared to HD-MSCs, despite no difference in terms of morphology and cell differentiation capacity (56). Moreover, MPN-MSCs exhibit an altered expression of several genes involved in cell differentiation and migration (56), such as the MYADM and Angiopoietin-1. Differences between HD- and MPN-MSCs have been found also in the cytokine profile. In fact, MSCs secrete a wide range of soluble factors (VEGFA, CXCL12, ILs) to support regenerative processes and perform immunomodulatory properties on NK cells, lymphocytes and macrophages (57). For example, Activin A, a cytokine involved in inflammation and erythropoiesis, has been reported to induce high grade marrow fibrosis in some MPN patients (8). Another example is provided by secretion of TNF-α, IL-10, and TGF-β, known to reduce the number and function of anti-leukemic cytotoxic T lymphocytes. Moreover, cytokines such as ILs (IL-2, IL-4, IL-6, IL-8, IL-10), GM-CSF, and TGF-β released by MSCs alter the innate and adaptative immune cell activation status to favour tumour development, and progression (58).

MSCs exert a pro-tumorigenic function by favouring the survival and differentiation of mutated haematopoietic precursors (59) through poorly defined mechanisms. Among them, the increased release of extracellular vesicles (EVs) is associated with inflammation and thrombosis, and sustained malignant haematopoiesis (60, 61). Based on this, EVs could represent biomarkers of MPN onset (62), as crucial players in regulating tumour microenvironment through the education of key processes including vascular reactivity, angiogenesis, chemoresistance and immunity. Due to their biocompatibility, small size, ability to cross biological membranes and capacity to target specific cells, EVs also represent a promising new approach for drug delivery (63). Indeed, EVs have been studied as cargo of various oligonucleotides of natural and synthetic origin like Paclitaxel, Doxorubicin and phytochemicals (64). Another mechanism exploited by MPN-MSCs could be an exaggerated activation of the pro-inflammatory NF-kB pathway, leading to cytokine release, and proliferation and maintenance of the mutated HSCs and myeloid and lymphoid precursors (65, 66).

Interestingly, MSCs may also have an anti-tumorigenic function, as demonstrated by decreased *in vitro* TC proliferation and reduced *in vivo* tumour growth, through mechanisms including cell cycle arrest and inhibition of angiogenesis (67); this MSC behaviour has been better characterized in the framework of solid tumours like breast and lung cancers (68, 69). Additional evidence of MSC anti-tumorigenic function is the *in vivo* expansion of MPN-HSC and accelerated MPN progression observed in a murine model of the disease after Nestin^+^ MSC depletion and consequent reduction of MSC-derived CXCL12. On the contrary, prevention of Nestin^+^ MSC loss blocks MPN progression by indirectly reducing the number of leukemic stem cells (70).

Overall, this points to a delicate balance between opposite properties of the MSC population, with implications for therapy.

This “Janus” attitude of MSCs with respect to MPN may remind the well-known behaviour of M1/M2 macrophages in the framework of solid tumours (71). Further research will strengthen this intriguing parallelism.

### A Role for MSCs in MDS

Most mutations found in MDS patients do not confer an obvious selective advantage to HSPCs that justify the clonal dominance. Indeed, MDS cells have been shown to alter the BMME and exploit cell extrinsic factors to maintain a selective advantage over non-mutated cells, as reviewed elsewhere (72, 73). Moreover, the altered microenvironment is harmful also to normal HSCs, thus negatively affecting the outcome of allogeneic HSPC transplantation.

Alterations of the BMME in MDS include disruption of the BM architecture and higher bone fragility. The mechanism underlying bone defects in MDS has not been fully clarified. For example, despite MSCs are altered in MDS and display recurrent mutations when expanded *ex vivo*, they are not clonally mutated *in vivo* (7). One intriguing explanation comes from an MDS murine model, in which delayed bone mineralization by OBs, due to increased levels of FGF-23, has been recently demonstrated (74). However, bone defects may also arise from functional impairment at the MSC level, including altered differentiation potential and cytokine production. Indeed, MSCs from MDS patients (MDS-MSCs) display increased *in vitro* adipogenic differentiation due to reduced DLK1 expression (75), likely at the expenses of the osteogenic potential. Functional inhibition of MSCs in MDS, leading to defective osteogenic differentiation capacity, is also mediated by TGF-β, present at increased levels in the MDS BMME (50), which cause abnormal gene expression of PITX2, HOXB6 and TBX15, leading to phenotypic and functional deficits (76).

In addition, MDS-MSCs have reduced HSC supporting capacity, as demonstrated by significantly lower chimerism in xenograft models when co-injecting HSPCs with MDS-MSCs as opposed to HD-MSCs (77). Further alterations in the osteo-haematopoietic niche may occur because of pharmacological therapies. For example, treatment with Rigosertib, a novel multi-kinase inhibitor anti-cancer drug currently tested in clinical trials for MDS, has been demonstrated to cause deterioration of the haematopoietic-supporting ability of MDS-MSCs, as shown by reduced number of colony-forming units, especially in the monocytic lineage, in a co-culture setting. In addition, Rigosertib impairs MDS-MSCs viability through microtubule destabilization and mitosis disruption, and decreases bone mass in a murine model of the disease (9).

An MDS-MSC driven mechanism has been shown to induce an immunosuppressive function in monocytes, which acquire an MDSC phenotype and suppress NK cell function (78). This in turn favours survival of MDS-HSPCs, although evidence in patients is lacking at present.

Increased amounts of BM Tregs, which have an immunosuppressive role, in high-risk compared to low-risk MDS patients has been recently described (79). BM Tregs directly affect the HSC supporting ability of BM MSCs. Whether Tregs from MDS patients affect MDS course by altering MSC function remains to be determined (80).

## The Bone-BMME: A Tumour Cell Soil

In line with the “seed and soil” concept for metastases establishment, MSCs are an important “soil” component since they can enhance the metastatic ability of TC by strengthening their motility and invasiveness. Moreover, they create a metastatic niche at secondary tumour sites (81, 82). MSCs have been reported to promote gastric (83), lung (84) and breast cancer (BCa) (85) growth and metastasis *via* stimulation of epithelial to mesenchymal transition (EMT). For instance, OPN release by TC was found to induce MSC production of the chemokine CCL5, which in turn promoted CCR5-mediated BCa cell motility, invasiveness, and metastasis. CCL5 was also reported to be secreted *in vitro* by human BM MSCs in response to osteosarcoma (86) and BCa cells. MSCs release also factors such as TGF-β, IL-10, NO, PGE2, and IDO, implicated in immunomodulation and thus relevant in creating a TC favourable environment (87).

BCa cells entry into the BM may be facilitated by MSCs through Tac-1 regulation of SDF-1α and CXCR4 (88). The subsequent establishment of TC within the BM results in a pathological cellular crosstalk disrupting bone homeostasis which is mainly controlled by the RANKL/RANK axis (89–91). Whether and how OCs contribute to the pre-metastatic niche and TC bone tropism is largely unknown. RSPO2 and RANKL, secreted by BCa cells as recruiting factors for OC precursors, have been demonstrated to bind their receptor LGR4 through an autocrine/paracrine loop and stimulate the production of DKK1, which acts on OC precursors to promote OC differentiation and pre-metastatic niche formation (92). RANKL signalling is harnessed also in other contexts. For example, in Multiple Myeloma (MM), a plasma cell malignancy developing in the BM, TCs have increased RANKL and decreased OPG expression, resulting in enhanced OC bone resorption and the development of lytic bone lesions (93). Moreover, CXCL12-expressing fibroblasts have been associated with a cancer-promoting phenotype in BCa and aggressive solid tumours (94), pointing to a role in bone metastases (95).

Not only solid, but also haematological cancers remodel the bone microenvironment and generate bone metastases. For example, patients with Adult T-cell leukaemia/lymphoma (ATL) may have widespread osteolytic lesions and hypercalcemia, and a novel ATL mouse model has been recently generated to dissect disease mechanisms and heterogeneity (96). Osteolytic and/or osteosclerotic lesions are present in Hodgkin’s Lymphoma patients, too (97). Moreover, scRNAseq analysis of the BM stroma in a murine model of AML has demonstrated that TCs impair mesenchymal osteogenic differentiation and deregulate the expression of CXCL12 and KITL (3). As another example, MM hijacks the BM niche through direct cell-cell interaction and MM-derived EV-mediated signalling; in this respect, the oncogenic NOTCH receptors have been recently identified as part of the MM-EV cargo with pro-tumorigenic effect (98).

Intercellular communication, even on the long range, has been demonstrated to occur also through diverse types of EVs including exosomes (99). Exosome-mediated PKM2 transfer from prostate cancer (PCa) cells into BMSCs has been shown to promote premetastatic niche formation. Specifically, exosome-derived PKM2 increased CXCL12 production by BMSC in a HIF-1α-dependent fashion, which in turn enhanced PCa seeding and growth in the BM. Accordingly, targeting this axis diminished exosome-mediated bone metastasis (100). Using a bone metastatic model of enzalutamide-resistant PCa, Henrich et al. demonstrated that BM myeloid cells *in vitro* and *in vivo* did uptake the EVs released by PCa, leading to activation of NF-κB signalling, enhancing OC differentiation, and decreasing myeloid TSP-1 expression. Reducing BM myeloid cell cholesterol, through systemic administration of nanoparticle mimic of native HDL, prevented the uptake of PCa EVs and, consequently, reduced metastatic burden by 77% (101).

RUNX2 and its regulated genes have been shown to facilitate the acquisition of osteomimetic features and enhance the bone metastatic potential of BCa cells, when overexpressed. Different EV proteins were identified mediating the specific recognition of tumour-derived EVs by OBs (CDH11) and the induction of the osteogenic premetastatic niche (ITGA5). These new markers were demonstrated to be responsible for the formation of a premetastatic niche, revealing a potential EV-based premetastatic niche blockage strategy (102).

## Therapeutic Implications

**Figure 1:**
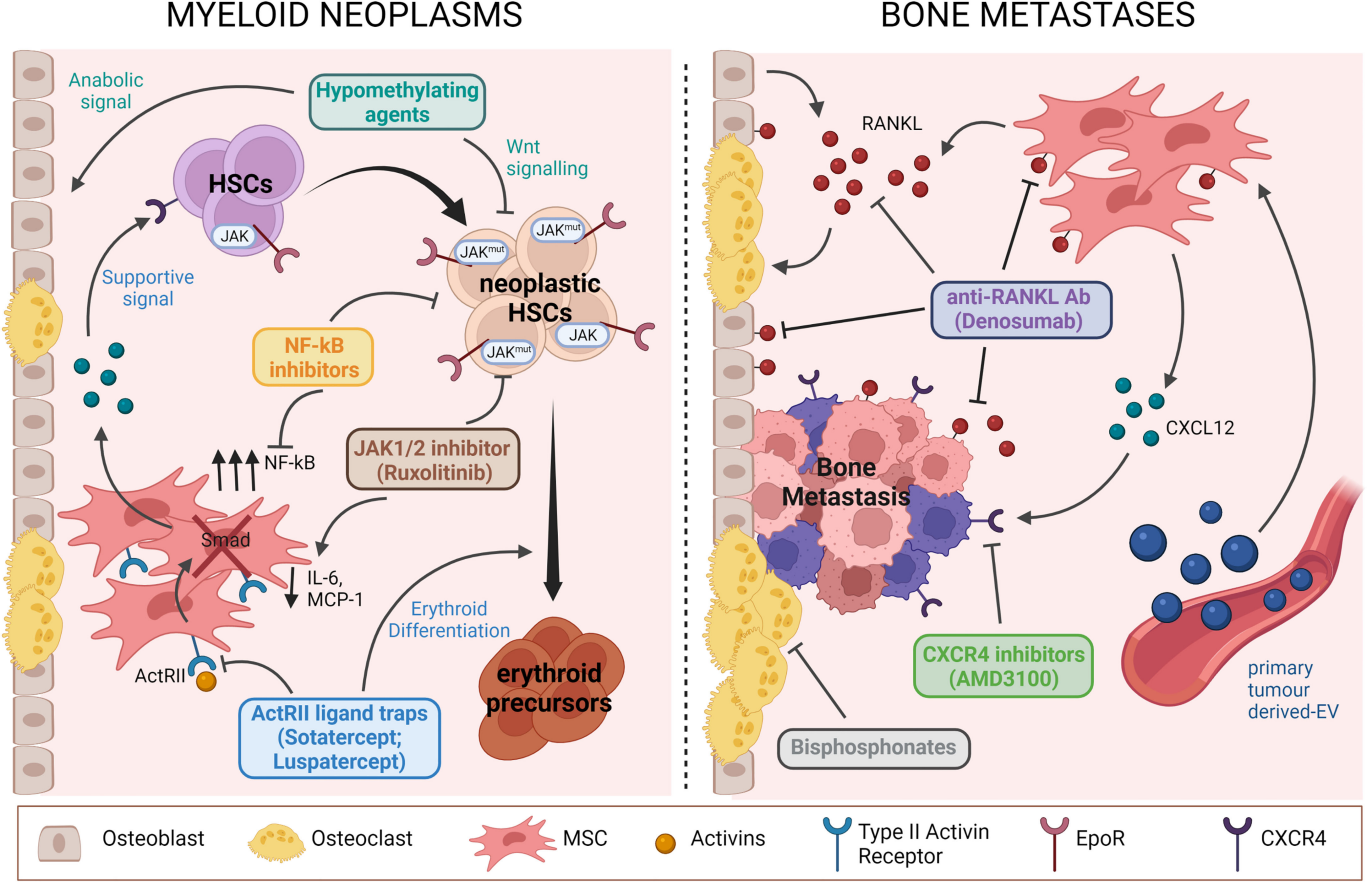
Schematic representation of pathological mechanisms involving BM niche components targeted by current therapies for myeloid neoplasms (we show the example of MPN) and bone metastases. Drugs acting both on BM niche cells and TCs are highlighted. Figure was created with BioRender.com.

Myelodisplastic and myeloproliferative disorders are characterized by vivid interactions with the osteo-haematopoietic niche. Thus, treatment strategies targeting not only malignant cells, but also the signalling pathways connecting both sides need to be developed to provide a more effective approach (**Figure 1**). The importance of the BMME is highlighted by the high rates of graft failure and relapses as well as the prolonged time to stabilize the engraftment in MDS patients after allogeneic HSPC transplantation; in fact, the prerequisite of a success stands within the appropriate milieu provided by the BMME and the crosstalk that must be re-built with the haematopoietic cells. In this perspective, for example, the hypomethylating agents Azacytidine and Decitabine are effective not only on the leukemic clone through Wnt signalling inhibition, but also on bone cells improving bone metabolism and favouring bone formation (103, 104). Additionally, Azacitidine in combination with Magrolimab (anti-CD47 antibody) and APR-246 (Eprenetapopt) are exploited in high-risk MDS patients including those with TP53 mutations, which have a complete remission rate lower than 20% with the standard-of-care Azacitidine therapy and poor prognosis. Mechanistically, APR-246 covalently binds to mutant p53 leading to its thermodynamic stabilization, thus shifting the equilibrium toward a functional conformation restoring its activity (105). In a phase Ib/II study, combination treatment with Eprenetapopt and Azacitidine is well-tolerated yielding high rates of clinical response and molecular remissions in patients with *TP53*-mutant MDS and oligoblastic AML (106).

Another example of treatment affecting both compartments is ACE-11 (Sotatercept), an activin receptor IIA (ActRIIA) ligand trap, which binds activin and other ligand of the TGF-β family thus interfering with the SMAD pathway (107). Sotatercept influences differentiation of erythroid progenitors or precursors probably by modulating factors of the BM niche (108). Indeed, stromal cells showed alterations in the expression of various important genes and cytokines in response to the drug (109). For example, several secreted proteins with relevance for the regulation of erythropoiesis were upregulated (e.g., IGFBP2, angiotensin II, BMP6, TSP1) or suppressed (e.g., VEGFA, OSM, BMP2) in response to ACE-011 treatment. The analogous ACE-536 (Luspatercept) targets preferentially GDF-8 and GDF-11, corrects the anaemia associated with ineffective erythropoiesis in the NUP98-HOXD13 murine model (110) and has been recently shown to reinstate SDF-1-mediated haematopoietic support by MSCs, thus restoring ineffective haematopoiesis (10). Importantly, both drugs promote maturation of late-stage Epo-independent erythroid precursors and co-treatment with Epo-induced synergistic responses suggesting their use for the treatment of MDS-related anaemia (108), as well as for concomitant alterations of the osteo-haematopoietic niche. In fact, Luspatercept was recently approved for the treatment of β-thalassemia (111) and for MDS low-risk patients with ring sideroblasts who have failed or are ineligible for erythropoiesis-stimulating agents (112).

An alternative strategy to interfere with the niche in MDS could be to counteract the iron overload by means of exogenous hepcidin, transferrin, hepcidin analogues and signalling agonists, since evidence in patients suggested that iron chelation could improve erythropoiesis (113).

The MSC population could be also influenced by Ruxolitinib, a drug used to treat MPN. Ruxolitinib is a JAK1/2 inhibitor that reduces JAK-STAT signalling, which is altered in MPN cells due to the presence of somatic mutations in JAK2 (JAK2^V617F^), CALR or MPL. The drug acts also on MSCs, by altering the expression level of fibrosis- and HSC maintenance-associated genes (such as *LOXL2*, *SPARC* and *ADAMTS4*, on one hand; and *CDH2*, *CXCL12* and *ANGPT1*, on the other) and by modifying the cytokine profile, reducing MCP-1 and IL-6 secretion (114).

Moreover, based on the reported hyperactive NF-kB signalling in MPN-MSCs, this pathway could serve as a target in a combined therapeutic approach against haematological malignancies, using for example a NF-kB inhibitor such as Bortezomib or Carfilzomib (66).

For what pertains to bone metastases, treatment is aimed at preventing disease progression and alleviating symptoms and may vary depending on the disease. The classical bone-targeting agents bisphosphonates and Denosumab (anti-RANKL antibody) have been shown to decrease the incidence of skeletal-related events in patients with MM and in those with bone metastases (regardless of the tumour type), but they do not replace the missing bone and, therefore, patients remain at risk of developing fractures, while the use of bone anabolic agents is not yet approved for routine clinical practice (115).

A wide range of agents studied in the last decade block OC bone resorption (116), including Everolimus (mTOR inhibitor), cathepsin K (a protease that degrades collagen during bone resorption) inhibitors (117), SRC tyrosine kinase inhibitors (118) and Cabozantinib (an inhibitor of receptor tyrosine kinases including VEGFR2 and MET) (119).

Emerging targets are the RSPO2/RANKL-LGR4 axis for inhibiting BCa bone metastasis (92), as well as metabolic factors like cholesterol homeostasis (101), that plays critical gate-keeping roles in regulating pro-metastatic signals by target cells at distant sites; both would motivate strategic diagnostic and therapeutic interventions aimed at preventing metastasis. Moreover, the exosome-induced CXCL12 axis could be another actionable pathway, based on the promising results in diminishing exosome-mediated bone metastasis (100). Notably, preventing CXCL12-CXCR4 interaction with the CXCR4 inhibitor Plerixafor (AMD3100) disrupted MM cell contacts with the BMME, thus leading to MM cell mobilization into the circulation (120).

## Conclusions and Perspectives

In conclusion, up-to-date extremely powerful technologies have been increasingly unveiling the kaleidoscopic nature of BM niches and their changes in pathologic conditions. In this microenvironment, MSCs are a key component and attract specific interest for therapeutic purposes, even though thus far successful applications in the clinic are limited, while strategies to exploit their plasticity *in situ* could be explored to bring results closer to expectations. In the tumour setting, future research should better dissect the mechanisms underlying altered MSC function. A pro- and anti-tumorigenic function has been demonstrated for MSCs with respect to MPN cells. To the best of our knowledge, no evidence of a similar mechanism in MDS cells is present in the literature, while it would be worth investigating, particularly for therapeutic purposes.

In the framework of bone metastases, whether TC infiltration elicits long lasting effects on the BM resident cells and whether the BMME remains dysfunctional even after depletion of TC from the metastatic site, are open questions. In a translational perspective, a better understanding of the impact of TC infiltration on the BM milieu could reveal better therapeutic targets. A mechanism of cell-cell communication raising much interest lastly is the crosstalk mediated by EVs. Tumour-derived EV are potent mediators of pre-metastatic niche formation due to their pro-malignant molecular cargo and their propensity to target specific cell types, thus engineering EVs as drug carriers for targeted therapy is an attractive option. Last, combination of the most effective therapies that address different mechanisms, depending on the disease, is likely to be superior to any single therapy.

## Author Contributions

VG wrote the Abstract and the “*A role for MSCs in MPN*” chapter. LC and FF wrote the “*BM Niches as Tumour Cell Factory in Acquired Blood Disorders: A Focus on MPN and MDS*" and “*A role for MSCs in MDS*” chapters. And also: All the authors contributed to the “*Conclusions and Perspectives*” chapter. CN wrote the “*The Bone-BMME: a tumour cell soil*” and “*Therapeutic implications*” chapters. CS wrote the “*Introduction*” and “*Composition of BM niches*” chapters. All the authors contributed to the “*Discussion and perspectives*” chapter. LC generated the figure. All the authors contributed to the “*Conclusions and perspectives*” chapter. All authors contributed to the article and approved the submitted version.

## Funding

This work was partially supported by the Italian Ministry of Health (grant RF-2018-12367680 to CS).

## Conflict of Interest

The authors declare that the research was conducted in the absence of any commercial or financial relationships that could be construed as a potential conflict of interest.

## Publisher’s Note

All claims expressed in this article are solely those of the authors and do not necessarily represent those of their affiliated organizations, or those of the publisher, the editors and the reviewers. Any product that may be evaluated in this article, or claim that may be made by its manufacturer, is not guaranteed or endorsed by the publisher.

## Abbreviations

CXCL12, C-X-C Motif Chemokine Ligand 12; SP7, Osterix; LepR, Leptin Receptor; SCF, Stem Cell Factor; IL-6, Interleukin-6; FGF2, Fibroblast Growth Factor 2; NLRP3, NLR Family Pyrin Domain Containing 3; JAK2, Janus Kinase 2; MPL, Myeloproliferative Leukemia, Thrombopoietin Receptor; CALR, Calreticulin; STAT, Signal Transducer and Activator of Transcription; TLR, Toll Like Receptor; IL-1β, Interleukin-1β; IFN, Interferon; VEGFA, Vascular Endothelial Growth Factor A; MYADM, Myeloid Associated Differentiation Marker; GM-CSF, Granulocyte-Macrophage Colony-Stimulating Factor; TGF-β, Transforming Growth Factor Beta; NF-kB, Nuclear Factor Kappa B; WNT, Wingless-type; AKT, Serine/Threonine Kinase 1; FGF-23, Fibroblast Growth Factor 23; DLK1, Delta Like Non-Canonical Notch Ligand 1; CCR5, C-C chemokine receptor type 5; CCL5, C-C Motif Chemokine Ligand 5; IL10, Interleukin-10; NO, Nitric Oxide; PGE2, Prostaglandin E2; IDO, Indoleamine 2,3-dioxygenase; SDF-1α, Stromal cell-Derived Factor 1α; CXCR4, C-X-C Motif Chemokine Receptor 4; RANKL, Receptor Activator of Nuclear Factor kappa B Ligand; RANK, Receptor Activator of Nuclear Factor kappa B; OPG, Osteoprotegerin; RSPO2, R-spondin 2; LGR4, Leucine Rich Repeat Containing G Protein-Coupled Receptor 4; DKK1, Dickkopf 1; PKM2, Pyruvate Kinase M2; TSP-1, Thrombospondin-1; RUNX2, Runt-related transcription factor 2; CDH11, Cadherin 11; ITGA5, integrin subunit alpha 5; FBXW7, F-Box And WD Repeat Domain Containing 7; PTEN, Phosphatase And Tensin Homolog; GSK3, Glycogen Synthase Kinase 3; IGFBP2, Insulin Like Growth Factor Binding Protein 2; BMP6, Bone Morphogenetic Protein 6; TSP1, Thrombospondin 1; OSM, Oncostatin M; BMP2, Bone Morphogenetic Protein 2; GDF-8, Growth/Differentiation Factor 8; GDF-11, Growth/Differentiation Factor 11; MCP-1, Monocyte Chemoattractant Protein-1; LOXL2, Lysyl-Oxidase 2; SPARC, Secreted Protein Acidic and Cysteine Rich; ADAMTS4, ADAM Metallopeptidase with Thrombospondin type 1 motif 4; CDH2, Cadherin 2; MET, Mesenchymal Epithelial Transition; TPO, thrombopoietin; PTN, pleyotropin; ANGPT, Angiopoietin; JAG1, Jagged-1; PGI_2_, Prostacyclin/prostaglandin I2; OPN, Osteopontin; VCAM1, vascular cell adhesion molecule 1.

## Glossary

**Table:**

CXCL12	C-X-C Motif Chemokine Ligand 12
SP7	Osterix
LepR	Leptin Receptor
SCF	Stem Cell Factor
IL-6	Interleukin-6
FGF2	Fibroblast Growth Factor 2
NLRP3	NLR Family Pyrin Domain Containing 3
JAK2	Janus Kinase 2
MPL	Myeloproliferative Leukemia, Thrombopoietin Receptor
CALR	Calreticulin
STAT	Signal Transducer and Activator of Transcription
TLR	Toll Like Receptor
IL-1β	Interleukin-1β
IFN	Interferon
VEGFA	Vascular Endothelial Growth Factor A
MYADM	Myeloid Associated Differentiation Marker
GM-CSF	Granulocyte-Macrophage Colony-Stimulating Factor
TGF-β	Transforming Growth Factor Beta
NF-kB	Nuclear Factor Kappa B
WNT	Wingless-type
AKT	Serine/Threonine Kinase 1
FGF-23	Fibroblast Growth Factor 23
DLK1	Delta Like Non-Canonical Notch Ligand 1
CCR5	C-C chemokine receptor type 5
CCL5	C-C Motif Chemokine Ligand 5
IL10	Interleukin-10
NO	Nitric Oxide
PGE2	Prostaglandin E2
IDO	Indoleamine 2,3-dioxygenase
SDF-1α	Stromal cell-Derived Factor 1α
CXCR4	C-X-C Motif Chemokine Receptor 4
RANKL	Receptor Activator of Nuclear Factor kappa B Ligand
RANK	Receptor Activator of Nuclear Factor kappa B
OPG	Osteoprotegerin
RSPO2	R-spondin 2
LGR4	Leucine Rich Repeat Containing G Protein-Coupled Receptor 4
DKK1	Dickkopf 1
PKM2	Pyruvate Kinase M2
TSP-1	Thrombospondin-1
RUNX2	Runt-related transcription factor 2
CDH11	Cadherin 11
ITGA5	integrin subunit alpha 5
FBXW7	F-Box And WD Repeat Domain Containing 7
PTEN	Phosphatase And Tensin Homolog
GSK3	Glycogen Synthase Kinase 3
IGFBP2	Insulin Like Growth Factor Binding Protein 2
BMP6	Bone Morphogenetic Protein 6
OSM	Oncostatin M
BMP2	Bone Morphogenetic Protein 2
GDF-8	Growth/Differentiation Factor 8
GDF-11	Growth/Differentiation Factor 11
MCP-1	Monocyte Chemoattractant Protein-1
LOXL2	Lysyl-Oxidase 2
SPARC	Secreted Protein Acidic and Cysteine Rich
ADAMTS4	ADAM Metallopeptidase with Thrombospondin type 1 motif 4
CDH2	Cadherin 2
MET	Mesenchymal Epithelial Transition
TPO	thrombopoietin
PTN	Pleiotropin
ANGPT	Angiopoietin
JAG1	Jagged-1
PGI_2_	Prostacyclin/prostaglandin I2
OPN	Osteopontin
VCAM1	vascular cell adhesion molecule 1
